# Traffic and combustion emission controls leave cooking as a dominant local source of primary PM_1_ in megacities

**DOI:** 10.1038/s41612-026-01583-6

**Published:** 2026-10-23

**Authors:** Ben Langford, Chiara, F. Di Marco, James M. Cash, James D. Allan, Zixuan Cheng, Hugh Coe, Delphine K. Farmer, Pingqing Fu, Ranu Gadi, Bhola Ram Gurjar, Jose L. Jimenez, Joel Kimmel, Dantong Liu, Tuhin Kumar Mandal, Neil J. Mullinger, Gavin J. Phillips, Ernesto Reyes-Villegas, Rutambhara Joshi, Shivani Shivani, Eiko Nemitz

**Affiliations:** 1 UK Centre for Ecology & Hydrology, Edinburgh Research Station, Bush Estate, Penicuik, UK; 2 School of Earth and Environmental Sciences, The University of Manchester, Manchester, UK; 3 National Centre for Atmospheric Science, The University of Manchester, Manchester, UK; 4 Department of Chemistry, Colorado State University, Fort Collins, CO USA; 5 Institute of Atmospheric Physics, Chinese Academy of Sciences, Beijing, China; 6 Department of Applied Sciences and Humanities, Indira Gandhi Delhi Technical University for Women, Delhi, India; 7 Department of Civil Engineering, Indian Institute of Technology, Roorkee, India; 8 Cooperative Institute for Research in Environmental Sciences (CIRES) and Department of Chemistry, University of Colorado, Boulder, CO USA; 9 TOFWERK AG, Thun, Switzerland; 10 Aerodyne Research Inc, Billerica, MA USA; 11 Zhejiang University, Hangzhou, China; 12 CSIR National Physical Laboratory, New Delhi, India; 13 Department of Natural Sciences, University of Chester, Thornton Science Park, Chester, UK; 14 Present Address: School of Earth System Science, Tianjin University, Tianjin, China; 15 Present Address: Maastricht University, Maastricht, Limburg Netherlands; 16 Present Address: Tecnologico de Monterrey, Escuela de Ingeniería y Ciencias, Av. General Ramon Corona 2514, Zapopan, Jalisco Mexico; 17 Present Address: UK Centre for Ecology & Hydrology, Maclean Building, Wallingford, UK

**Keywords:** Climate sciences, Environmental sciences, Environmental social sciences

## Abstract

Megacities frequently suffer from poor air quality, with PM_2.5_ concentrations routinely exceeding WHO guidelines and elevating the risk of cardiovascular and respiratory disease. Air quality legislation typically targets primary emissions from traffic, a dominant urban source of PM₂.₅. Here, we present direct, chemically resolved quantification of PM₁ emissions from three contrasting megacity neighbourhoods—London, Beijing and Delhi—revealing the magnitude and diversity of urban sources by applying a novel source attribution approach to eddy-covariance flux measurements using aerosol mass spectrometry. In Delhi, traffic and combustion dominate primary PM₁ emissions. In contrast, cooking is the largest source during mealtimes in London and Beijing, contributing up to 83% of midday emissions and 87% in the early evening in London, and up to 61% and 76%, respectively, in Beijing. These findings suggest that air quality legislation in London and Beijing has effectively reduced non-refractory transport and combustion emissions, allowing cooking to emerge as the largest local source of primary non-refractory PM₁. Although cooking is not included in national, European or global emission inventories, recent estimates from the London Atmospheric Emissions Inventory align well with our observations. Together, these results show that the sources dominating primary non-refractory PM₁ emissions differ from those controlling ambient PM_1_ mass. Although cooking is now the leading primary source in London and Beijing, further reductions in urban PM₁ will depend on mitigating secondary formation processes.

## Introduction

More than half the world’s population live in cities and this figure is projected to rise to 70% by 2050^1^. This rapid urbanisation has led to the emergence of megacities, metropolitan areas with populations exceeding 10 million, of which there are currently 34 worldwide^2^. The extremely high population densities in these urban centres (ranging from 4000 to over 10,000 per km^-2^)^3^ are often associated with significant environmental challenges, particularly poor air quality^4–6^. Air pollution remains the largest environmental risk to public health, contributing to an estimated 8.8 million premature deaths globally each year^7^. Of particular concern is exposure to fine particulate matter(PM_2.5_), which, due to its small size ( < 2.5 μm), can penetrate deep into the lungs and cross into the bloodstream^8^. PM is known to cause and exacerbate cardiovascular and respiratory diseases and has been linked to cancers of the mouth^9^, neck and lungs^10^, and is increasingly associated with adverse mental health outcomes^11^.

In response, the World Health Organization (WHO) updated its air quality guidelines in 2021, recommending that PM_2.5_ concentrations not exceed 15 µg/m^3^ over any 24-hour period and 5 µg/m^3^ on an annual average basis—standards that remain in place as of 2026. However, in megacities where transport, industry, and energy production are densely concentrated, meeting these limits remains a formidable challenge for local and national authorities.

Analysis of PM concentrations, chemical composition, and roadside increments have significantly advanced our understanding of the mix of urban emission sources^12–14^. A recent systematic review^15^ of measurements from 51 countries found that, on average, traffic-related black carbon and organic particles account for 25% of urban PM_2.5_, followed by domestic fuel combustion (20%), industrial activities including power generation (15%), and dust or sea salt (18%). Notably, 22% of PM_2.5_ was attributed to sources of unspecified human origin, underscoring the complexity and incomplete understanding of urban particulate sources^15^.

In the European Union, vehicle emissions have been a major focus of air quality legislation since the introduction of Euro 1 standards in 1992 (Euro 1)^16^. These regulations have become progressively stricter, culminating in the rollout of Euro 7 standards in 2025, which introduce tighter limits on ultrafine particles, brake and tyre wear, and real-world emissions testing. In cities like London, additional measures such as congestion charges and ultra-low emission zones (ULEZ) have been implemented to further reduce pollution. Yet, PM_2.5_ levels remain a serious public health concern. In 2022, 96% of Europe’s urban population was exposed to PM_2.5_ concentrations exceeding the WHO’s annual guideline of 5 µg m^3^, despite only 2% of monitoring stations breaching the EU’s older limit of 25 µg m^-3^^17^. In North America, recent work has shown that non-traditional sources of PM_2.5_, including cooking, transported wildfire smoke, and anthropogenic chemical products, are now dominating summertime particulate air pollution in New York^18,19^.

To meet air quality targets, more ambitious and costly interventions are now required. As an example, in the UK and European Union, legislation has been introduced to phase out the sale of new petrol and diesel vehicles by 2030 (2035 for EU). While primarily motivated by climate commitments and the drive towards NetZero, this transition is expected to significantly reduce PM_2.5_ by eliminating tailpipe exhaust emissions. However, the full benefits will take years to materialise, and few legislative tools remain to further reduce traffic-related emissions in the short term. A more detailed understanding of primary sources and their relative contributions is therefore essential to guide future policy and implement effective interventions.

Micrometeorological flux measurements offer a powerful tool for directly quantifying pollutant emissions, providing a top-down verification of emission inventories across a range of pollutants^20–27^. These measurements yield high-resolution spatial and temporal data that are critical for refining source attribution. Despite their potential, direct flux measurements of PM mass above cities remain rare^28,29^, with most studies focusing on particle number fluxes (e.g. # particles m^-2^ s^-1^)^30–32^. While particle number fluxes provide value, they lack the chemical resolution needed for detailed source apportionment. However, recent technological advances have begun to enable chemically-resolved flux measurements of sub-micron aerosol^33–36^.

In this study, we present the first direct flux measurements of chemically resolved submicron particulate matter (PM_1_) at city centre locations above three major megacities: London, Beijing, and Delhi, each integrating over a mix of commercial, institutional, residential and traffic sources. These cities differ markedly in population, vehicle fleets, emission sources, and air quality management strategies. By combining flux measurements with novel source apportionment techniques, we provide direct quantification of individual PM_1_ sources and identify opportunities for targeted legislative interventions. While the broader policy and health context centres on PM₂.₅, the aerosol mass spectrometer (AMS) instruments deployed here quantify non-refractory PM₁, which typically represents the majority of urban PM_2.5_ mass and certainly contains most secondary inorganic aerosol and organic aerosol components responsible for urban fine particulate pollution. This size fraction also provides the chemical resolution required for source attribution, particularly of primary organic aerosol sources. However, it does not capture refractory contributions to PM_2.5_, including mineral dust, sea salt and non-exhaust traffic emissions such as brake, tyre and road wear. Previous comparisons of co-located PM₁ and PM₂.₅ ACSM/AMS measurements have shown that, whilst in very polluted environments a minor fraction of the secondary aerosol components can be contained in particles larger than 1 µm the bulk chemical composition and source contributions are typically very similar, supporting the use of non-refractory PM₁ as a chemically informative measure of urban fine particulate pollution^37^.

## Results

Measurements were conducted from the Institute of Atmospheric Physics tower in Beijing during winter 2016 and summer 2017, the Indira Gandhi Delhi Technical University for Women in Old Delhi during autumn 2018 and the BT Tower in central London during summer and winter 2021. Sampling heights were 102 m, 33 m and 190 m above street level, respectively. These heights were selected to ensure measurements were made above the heterogeneous urban canopy and therefore represented integrated neighbourhood-scale emissions, while also reflecting the availability of suitable tower infrastructure in each city (see Supplementary Information for details).

Although the resulting flux footprints differed between cities, all measurements were designed to provide representative neighbourhood-scale fluxes rather than identical source areas. Consequently, comparisons should be interpreted as contrasts between integrated urban source mixtures. The London footprint encompassed a dense commercial city centre with major roads, railway stations and a large number of restaurants and commercial kitchens. The Beijing footprint represented a mixed residential and commercial district between the third and fourth ring roads, containing major road corridors and numerous restaurants within a few hundred metres of the tower. The Delhi footprint was strongly influenced by major transport infrastructure, including busy roads, a railway station and a large bus terminal. Further details of the site environments and footprint characteristics are provided in the Supplementary Information.

**Fig. 1:**
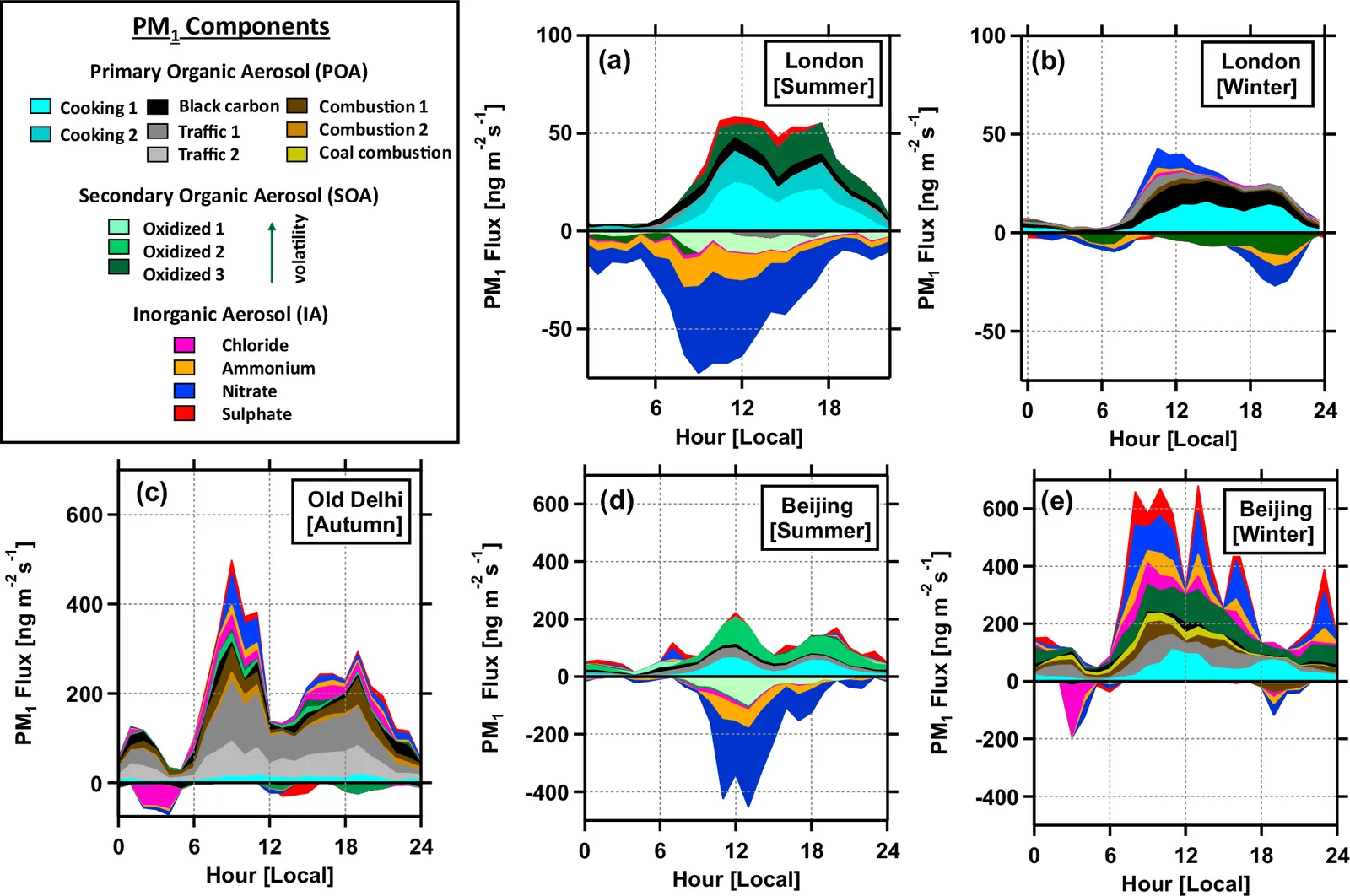
Diurnal source-resolved PM₁ fluxes differ substantially between London, Beijing and Delhi. Average diurnal fluxes of chemically resolved submicron particulate matter (PM₁) measured above London during summer (**a**) and winter (**b**), Delhi during autumn (**c**), and Beijing during summer (**d**) and winter (**e**). Stacked areas show the contributions of individual aerosol components and source factors to the measured PM₁ flux. Primary organic aerosol (POA) factors include cooking aerosol (Cooking 1 and Cooking 2), traffic-related aerosol (Traffic 1 and Traffic 2), combustion aerosol (Combustion 1 and Combustion 2), coal combustion aerosol and black carbon (BC). Secondary organic aerosol (SOA) factors comprise Oxidised 1, Oxidised 2 and Oxidised 3, ordered from the most volatile (Oxidised 1) to the least volatile (Oxidised 3). Inorganic aerosol components include chloride, ammonium, nitrate and sulphate. Positive values indicate net emission to the atmosphere and negative values indicate net deposition. Colours correspond to the source categories shown in the legend panel.

Figure 1 presents top-down flux measurements of submicron particulate matter (PM_1_) from these three megacities. Emissions are categorised into non-refractory inorganic components, nitrate, sulfate, ammonium, and chloride, and organic aerosol, which is further resolved into source factors using positive matrix factorization (PMF)^38,39^ (Methods). These factors include both primary sources (traffic, solid fuel combustion, coal combustion, and cooking) and secondary oxidised components, which represent chemically processed material that is more difficult to attribute to a single source. Black carbon (BC) emissions in the PM_1_ size range, were also directly measured at each site using eddy covariance^40,41^.

Across all cities, the observed total PM_1_ fluxes were positive, indicating net emissions. However, in both Beijing and London during summer, several inorganic components and the less oxidised organic aerosol factor exhibited periods of deposition. This behavior is likely related to the semi-volatile nature of these species and is discussed further in Nemitz et al. (in preparation). Specifically, the apparent deposition does not reflect physical deposition to the surface, but rather an artefact of vertical partitioning: as semi-volatile aerosol particles descend toward the surface, they encounter a temperature gradient that promotes evaporation into their gas-phase precursors (e.g. NH_3_ and HNO_3_) below the measurement height. This phase change is interpreted as deposition by the flux system, even though the material is not removed to the surface

Despite these seasonal differences, emissions generally peaked during the mid-to-late morning, with a secondary enhancement in the late afternoon to early evening. However, both the magnitude and composition of emissions varied substantially between cities and seasons. The London diurnal profiles (Fig. 1a, b) were noticeably smoother than those observed in Beijing (Fig. 1d, e) and Delhi (Fig. 1c), exhibiting broad daytime enhancements rather than the pronounced peaks seen elsewhere. Similar behaviour has previously been observed for VOC fluxes measured above central London, where traffic-related emissions remained elevated throughout much of the day rather than displaying sharp rush-hour peaks, reflecting the sustained commercial and transport activity characteristic of the city centre^42^. London also exhibited comparatively weak inorganic aerosol fluxes, particularly during winter, indicating that direct emissions of inorganic PM₁ components are now relatively minor within the city centre.

Sulphate fluxes also showed clear differences between cities. In Beijing, sulphate emissions were evident in both seasons, with wintertime fluxes more than twice those measured during summer. Sulphate, treated here as a near-surface emission source, originates from combustion processes including vehicle exhaust and coal burning^43^. However, the presence of chloride emissions during winter only, a well-established tracer of coal combustion, suggests that coal was the dominant source of wintertime sulphate, while traffic played a comparatively minor role. This interpretation is supported by the very low sulphate fluxes observed in London, where ultra-low sulphur fuels minimise vehicle-related sulphur emissions and coal combustion sources are largely absent from the flux footprint. Similarly, chloride emissions were negligible in London throughout the measurement period. The contrast between the strong wintertime sulphate and chloride emissions observed in Beijing and their near absence in London supports the conclusion that coal combustion, rather than traffic, is the primary driver of seasonal sulphate variability.

**Fig. 2:**
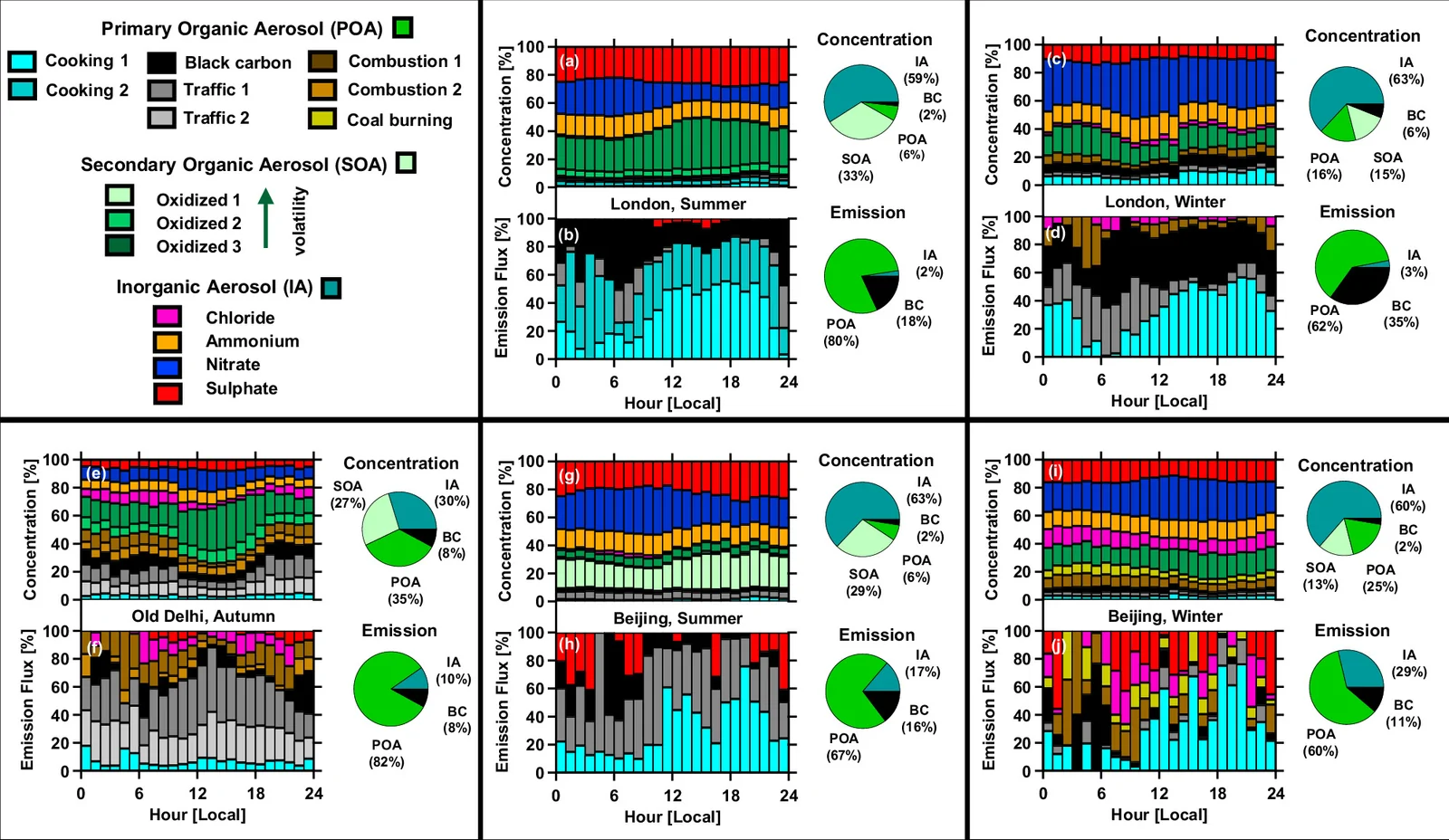
The relative importance of individual PM₁ sources differs between emissions and ambient concentrations. Panels (**a**), (**c**), (**e**), (**g**) and (**i**) show the fractional contribution of individual components to ambient PM₁ concentrations, while panels (**b**), (**d**), (**f**), (**h**) and (**j**) show the fractional contribution to primary PM₁ emissions. Results are shown for London in summer (**a**, **b**) and winter (**c**, **d**), Delhi (**e**, **f**), and Beijing in summer (**g**, **h**) and winter (i,j). Stacked area plots illustrate the average diurnal variation in source contributions. IA denotes inorganic aerosol, SOA denotes secondary organic aerosol, BC denotes black carbon and POA denotes primary organic aerosol. Fractions are expressed as percentages of total PM₁ concentration or total primary PM₁ emission flux. Colours correspond to the source categories shown in each panel.

Figure 2 shows the relative fraction of PM_1_ attributed to each source on both a concentration and emission basis over an average day. Although secondary inorganic and oxidised organic components indicate a net emission at most sites, we limit our comparison of emissions to the primary components, including sulphate and chloride, which are linked to traffic and coal combustion. For each site, we show the breakdown of BC, primary organic (POA), oxidised organic (OOA), and secondary inorganic aerosols (SIA) on both a concentration and flux basis.

The chemical composition of PM_1_ concentrations varied markedly between the three studied megacities, reflecting differences in local and regional emissions, as well as secondary processing. SIA dominated PM_1_ mass in London (Fig. 2a, c) and Beijing Fig. 2g, i), accounting for 59% and 63% of total PM_1_ in summer, and 63% and 60% in winter, respectively. In contrast, SIA constituted only 30% of PM_1_ in Delhi (Fig. 2e), highlighting the comparatively smaller relative role of secondary inorganic formation in this highly organic, combustion-driven environment.

Primary organic aerosol, typically emitted directly from sources such as traffic, biomass burning, and domestic combustion, showed a complementary seasonal pattern to OOA. In London and Beijing, POA contributed a relatively minor fraction of PM_1_ mass during summer (6%) but increased substantially in winter (16–25%), likely due to enhanced domestic heating, reduced atmospheric mixing and photochemical oxidation. In contrast, Delhi exhibited a consistently high POA fraction (35%), reflecting the dominance of traffic and combustion emissions.

OOA, which encompasses both SOA formed from atmospheric oxidation of volatile precursors and aged POA that has undergone chemical transformation, displayed the opposite seasonal trend. It accounted for a larger fraction of PM_1_ in summer (33% in London, 29% in Beijing) than in winter (15% and 13%, respectively), consistent with increased photochemical activity and oxidant availability and suppressed formation of volatile ammonium nitrate during warmer months. This seasonal contrast highlights the dynamic evolution of organic aerosol: while POA dominates under conditions of strong primary emissions and limited photochemistry, OOA formation is enhanced when sunlight and oxidants drive the transformation of both VOCs and existing organic particles. In Delhi, OOA contributed 27% to PM_1_ mass, suggesting a relatively even split between POA, OOA, and secondary inorganic aerosol (SIA), shaped by the city’s persistent emissions and intense photochemical environment.

When focusing on primary emissions, cooking was found to be a major contributor to PM_1_ in London (Fig. 2b, d) and Beijing (Fig. 2h, j), ranging between 38–86% of emissions at certain times of day. In Delhi (Fig. 2f), cooking emissions were less significant, averaging just 8% of daily primary emissions. Traffic-related emissions (traffic factor + black carbon) contributed 16–38% in London, 28–46% in Beijing, and 56% in Delhi. The lower wintertime contribution in Beijing likely reflects the influence of additional combustion sources, such as coal and biomass burning, which are less prevalent in summer. In London, contributions from coal and biomass combustion were minimal due to the city’s designation as a Smoke Control Area, which restricts the use of polluting solid fuels and unauthorised appliances. Despite this dominance in primary emissions, cooking contributes only a small fraction to ambient PM₁ mass (5–10%), highlighting a clear divergence between emission fluxes and concentration-based source attributions.

Black carbon emissions in London were lower than those observed in Delhi and comparable to summer fluxes in Beijing, but their relative contribution was high. Average BC fluxes measured from the BT Tower were 6.8 ng m⁻² s⁻¹ in summer and 13.3 ng m⁻² s⁻¹ in winter. In Delhi, BC fluxes reached 25.8 ng m⁻² s⁻¹, consistent with the dominance of combustion-related emissions, including road traffic and solid fuel use. Although diesel vehicles are restricted to nighttime operation in both Beijing and Delhi, the latter still exhibits higher BC emissions, likely due to less stringent emission controls and a greater prevalence of older vehicle technologies. In London, flux footprint analysis revealed that construction activity—particularly non-road mobile machinery (NRMM)—was a strong contributor to BC emissions during winter, with traffic dominating at other times. These findings are discussed in detail by Cheng et al.^41^.

### Comparison with Emission Inventories

**Fig. 3:**
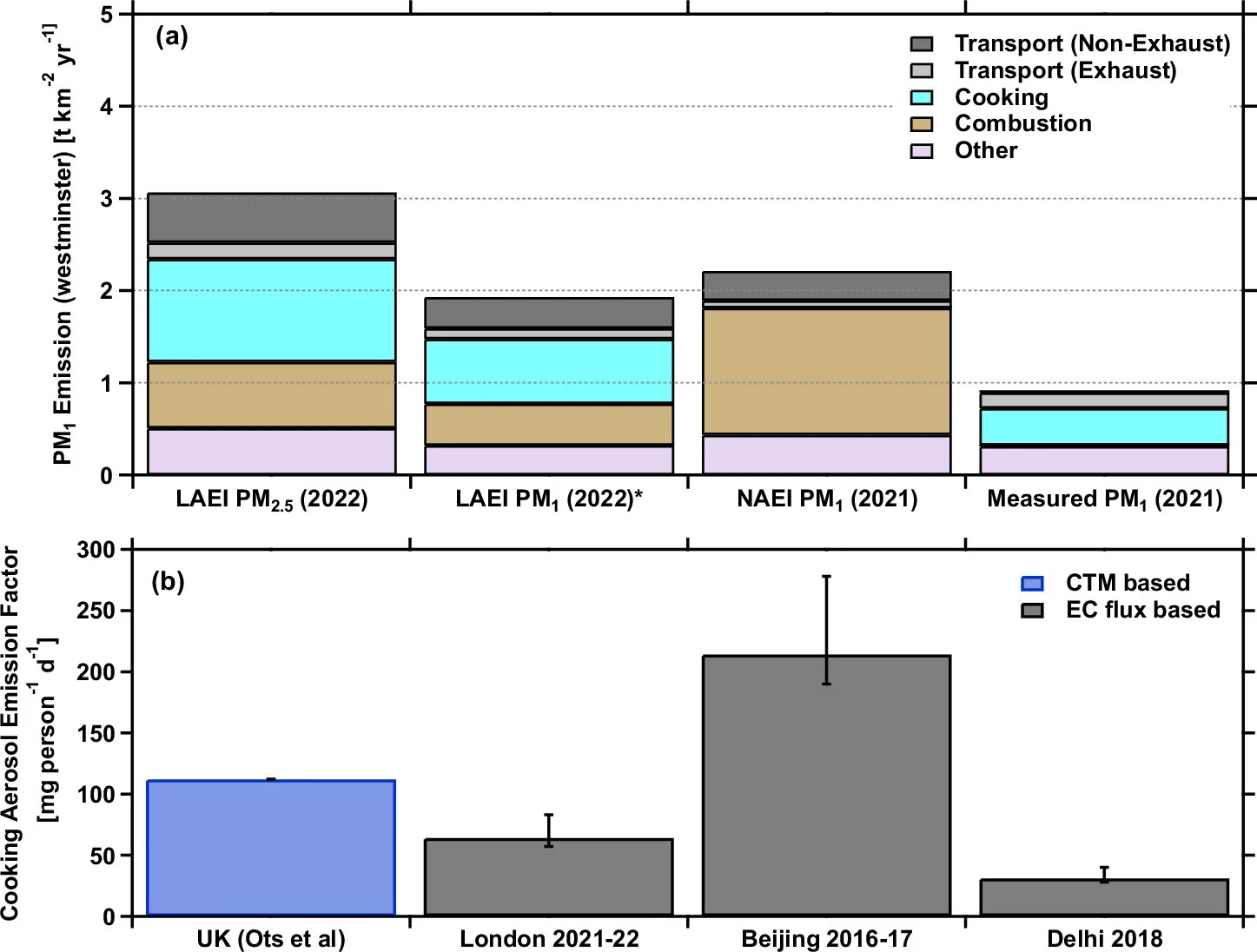
London PM₁ emission estimates derived from flux measurements are compared with national and regional inventories. **a** Compares PM emissions predicted for the London Borough of Westminster by the National Atmospheric Emissions Inventory (NAEI, 2022), the London Atmospheric Emissions Inventory (LAEI, 2022) and estimates derived from eddy-covariance (EC) flux measurements of non-refractory PM₁. Measured PM₁ fluxes were grouped into traffic (traffic factor plus black carbon), combustion (combustion factor), cooking (cooking aerosol factors) and other sources (nitrate, ammonium, chloride, sulphate and oxidised aerosol components). The LAEI reports PM₂.₅ emissions only; these values were scaled to PM₁ using the PM₂.₅:PM₁ ratio applied within the NAEI. **b** Shows cooking aerosol emission factors derived from measured fluxes and workday population estimates for London, Beijing and Delhi, together with the UK-wide cooking aerosol emission factor reported by Ots et al. (2016). Error bars represent uncertainty associated with the aerosol mass spectrometer relative ionisation efficiency (RIE = 1.4-2.5) and a ± 10% uncertainty in population density. The Ots et al. emission factor is model-derived but constrained using measured cooking aerosol concentration data.

Although emission inventories exist for Delhi and Beijing, they typically report PM₂.₅ or total particulate matter and do not provide the level of source-specific, non-refractory PM₁ detail required for direct comparison with flux measurements. To illustrate the value of such comparisons, Fig. 3 contrasts measured PM₁ fluxes in London with annual emissions from the UK’s National Atmospheric Emissions Inventory (NAEI, 2022, PM₁^44^) and the London Atmospheric Emissions Inventory (LAEI, 2022, PM₂.₅^45^) for the London borough of Westminster. This comparison is important because inventories underpin air quality modelling and policy decisions; validating them against real-world flux measurements helps identify potential biases in total emissions and source attribution. Long-term CO₂ flux measurements from the same site indicate that Westminster contributes approximately 65% of the average flux footprint, rising to over 80% under unstable meteorological conditions^26^. This makes Westminster a useful comparator for inventory validation.

Measured fluxes were extrapolated to annual totals by assuming that summer measurements represent eight months of the year and winter measurements the remaining four. For comparison, emissions were grouped into four broad categories: Transport (traffic factor + BC; NAEI Selected Nomenclature for Air Pollutant (SNAP) sectors 7–8; LAEI Transport), Combustion (combustion factor; SNAP sectors 1–3; LAEI Biomass, Heat & Power Generation, Waste), Cooking (COA factor; LAEI Domestic and Commercial Cooking), and Other (positive OOA and secondary inorganic aerosol components; SNAP sectors 5–6 and 9–11; LAEI all remaining sectors). The NAEI does not capture direct emissions from cooking, focusing instead on emissions associated with the fuel used to carry out the cooking – captured here under ‘combustion’. In contrast, the LAEI makes direct estimates of cooking emissions from both commercial and domestic sources. The total PM₁ emissions predicted by the NAEI were approximately twice the observed fluxes. For a consistent comparison with AMS measurements, we focus on exhaust emissions only, as the AMS captures non-refractory PM₁ and does not detect refractory particles associated with non-exhaust emissions (e.g. brake and tyre wear). This is particularly important for the transport sector, where non-exhaust sources account for around 75% of total emissions in the NAEI.

When restricting the inventories to exhaust emissions only (i.e. excluding non-exhaust sources shown in Fig. 3), both inventories appear to underestimate transport emissions relative to observations, with values 54% and 34% lower than measured for the NAEI and LAEI, respectively.

Similarly, the AMS combustion factor is most sensitive to biomass burning, particularly residential wood burning, and does not capture refractory emissions from gas boilers, industrial furnaces, or incinerators, which are included in SNAP sectors 1–3. This limits the comparability between AMS-derived combustion fluxes and inventory totals for combustion. Here very large differences are evident between measurements and inventory, suggesting that either combustion sources in these sectors predominantly emit refractory material not captured by AMS, or that their contributions are significantly overestimated in current inventories.

Cooking offers a more like-for-like comparison and shows the LAEI to overestimate observed cooking emissions by approximately 76%. Cooking emissions have only recently been incorporated into the LAEI, and methodologies are still evolving; however, the reported values fall within the range of observations. The LAEI is updated every few years, and the closest available year to our measurements was 2022. The higher values may reflect that, during our winter measurement period, the UK Government introduced Plan B measures in response to the emergence of the Omicron variant of COVID-19. These began on 8 December 2021 and included mandatory face coverings in most indoor public spaces, guidance to work from home where possible (from 13 December), and the requirement for NHS COVID Passes at large venues. Although hospitality venues remained open, the public were advised to limit social contact, which likely reduced restaurant and pub attendance, likely suppressing COA emissions relative to typical conditions. Overall, our measurements suggest cooking accounts for 45% of annual emissions, closely matching the relative contribution of cooking in the LAEI (When removing non-exhaust traffic emissions from the total) of 44%. In contrast, cooking emissions appear noticeably absent from the NAEI estimates.

Although cooking is now appearing in some regional and national level estimates, it is not accounted for in European or Global emission inventories. As a first order approximation, we estimated cooking aerosol emission factors for each of the three Megacities by dividing average daily emission totals by the average workday population densities, which better represent the daytime populations present within these city-centre environments than residential population densities, to arrive at values of 64, 214 and 31 mg person^-1^ d^-1^ for London, Beijing and Delhi, respectively. These values are shown in Fig. 3b alongside the estimated emission factor (112 mg person^-1^ d^-1^) proposed by Ots et al.^46^. The original emission factor reported by Ots et al. was 320 mg person⁻¹ d⁻¹, but has been adjusted here to harmonise the collection efficiency (CE = 1) and relative ionisation efficiency (RIE = 2) assumptions with those used in the present study. Ots et al. derived their value by combining annual average cooking organic aerosol (COA) concentrations measured by AMS at two London sites with workday population density data from the 2011 census. These AMS-derived COA concentrations were obtained via PMF and used to constrain emissions in the EMEP4UK atmospheric chemistry transport model (CTM). Notably, the lower London value may reflect reduced cooking activity in 2021 as the city recovered from COVID-19 lockdowns, with fewer commuters and restaurant patrons contributing to urban emissions. This behavioural shift, rather than population density alone, may have led to a genuine reduction in total cooking emissions during the measurement period

Overall, the promising agreement between flux-derived, population-based, and inventory estimates in London supports the validity of this scaling approach and highlights the potential for its application in other cities where direct flux measurements are unavailable. Failure to account for COA may lead to underestimation of primary PM_1_ in model simulations, particularly during midday and evening peaks, and contribute to discrepancies between observed and modelled urban PM concentrations.

## Discussion

Our measurements reveal that a substantial fraction of non-refractory primary PM_1_ emissions from developed urban centres originates from cooking, likely dominated by cooking oil emissions from shallow and deep frying in commercial settings^47^. Crucially, this dominance applies to emission fluxes rather than ambient concentrations: cooking contributes only a minor fraction of the urban background PM₁ mass as measured on urban flux towers, with secondary inorganic aerosol and oxidised organic components dominating observed loadings.

These emissions exhibit a pronounced diurnal pattern, with peaks at midday and early evening corresponding to mealtimes, during which cooking accounts for up to 86% and 76% of primary PM_1_ emissions in London and Beijing, respectively. In both cities, the flux footprint encompassed a large number of restaurants, which are likely responsible for the bulk of cooking-related emissions. In Beijing, cooking aerosol fluxes were substantially larger during winter than summer (40.6 and 23.2 ng m⁻² s⁻¹, respectively). While this may partly reflect seasonal differences in cooking activity, the measurements were also influenced by different source areas. Analysis of the same flux footprints by Squires et al. (2020) showed that wintertime measurements were influenced predominantly by areas northwest of the tower that contained commercial buildings and restaurants, whereas summertime measurements were more strongly influenced by areas to the east containing the Jingzang Highway. The larger wintertime cooking fluxes may therefore partly reflect the greater influence of restaurant-dominated source areas within the measurement footprint.

By contrast, traffic-related emissions were unexpectedly small, at times, contributing less than 10% of primary PM₁ in both London and Beijing—a finding that challenges common assumptions about the dominance of traffic as an urban PM source. This reflects the success of stringent vehicle emission controls and the transition towards cleaner fleets, particularly in central London and Beijing.

Further work is now needed to quantify direct emissions from suburban areas, where cooking PM is expected to be attributed mainly to domestic sources which may differ in magnitude due to variations in housing density, ventilation practices, and cooking styles. On a concentration basis, long-term monitoring of organic aerosol loadings at a suburban site in Athens have already revealed significant contributions of COA to annual averages (18% of total OA)^48^ - suggesting that domestic cooking is already an important source of PM_1_ outside of urban centres.

In Delhi, cooking emissions were less significant, contributing an average of 8% to daily primary PM₁ emissions. However, this should not be interpreted as evidence of low cooking activity. Absolute cooking aerosol fluxes in Delhi (9.7 ng m⁻² s⁻¹) were comparable to those observed in London, exceeding the average wintertime London value (6.6 ng m⁻² s⁻¹) and remaining only 35% lower than the summer value (15 ng m⁻² s⁻¹). While differences in urban morphology, measurement height and atmospheric stability influence the size, location and composition of the flux footprint, the comparatively small fractional contribution of cooking in Delhi appears primarily to reflect the much larger traffic and combustion emissions present within the Delhi source mix, which dominate primary aerosol emissions throughout much of the day. Consistent with this interpretation, the ratio of cooking aerosol to traffic-related emissions (HOA + BC) was substantially lower in Delhi (0.12) than in London (0.74-3.18) or Beijing (0.71-3.62). Differences in the type of urban matrix dominating the footprint may also contribute to the observed contrasts, with the London footprint encompassing a dense commercial city centre containing a large number of restaurants and commercial kitchens, while the Delhi footprint was more strongly influenced by transport corridors, residential areas, a major railway station and bus terminal. The locations and release heights of individual cooking exhausts were not characterised and represent an additional uncertainty in the precise quantitative comparison between cities. However, these site-specific factors principally affect the source areas represented within the flux footprint and are not expected to systematically account for the substantially lower relative contribution of cooking aerosol observed in Delhi.

Cooking is already recognised as the single largest source of PM in households and restaurants^49,50^, and our measurements suggest that these emissions are efficiently transported from indoor to outdoor environments. Once in the atmosphere, cooking-derived aerosol undergoes complex physical and chemical transformations. COA is rich in fatty acids such as oleic-, linoleic-, palitic- and stearic acid, which may self-assemble into nanostructures that slow oxidative ageing^51^, enhance water uptake, and prolong particle lifetimes from hours to days^52,53^. These transformations contribute to persistent primary PM and may influence cloud interactions via changes in hygroscopicity^54^ and CCN activity^55^. In addition to the directly emitted aerosol, cooking releases volatile and semi-volatile organic compounds that can undergo atmospheric oxidation to form secondary organic aerosol and contribute to photochemical ozone production^56^, linking cooking emissions to both particulate and gas-phase urban air pollution.

Cooking aerosol also poses a direct risk to health, with chefs shown to be at elevated risk of respiratory illness compared to the general population^50,57^. For this reason, in the UK and Europe, legislation governs the extraction of cooking fumes from domestic and commercial kitchens (e.g. EN 16282). However, it does not mandate filtration of the exhaust and the level of enforcement is low. As a result, these emissions remain unregulated and may offer a relatively low-cost intervention for reducing primary PM emissions in developed urban centres where cooking emissions are becoming an increasingly significant fraction of the primary source mix. In Delhi, such interventions are likely to be less effective due to poorly ventilated homes and a higher prevalence of outdoor cooking. Here, reducing emissions from traffic and combustion sources—which still dominate the local source mix—should remain the priority.

However, on a concentration basis—more relevant for assessing exposure—PM_1_ from cooking appears less dominant, contributing just 5–10% of ambient mass loadings, similar to traffic (5–18%). This apparent discrepancy arises because flux measurements quantify local emissions within the tower footprint, whereas ambient concentrations integrate the combined influence of local emissions, regional transport and atmospheric processing. As a result, sources that dominate local primary emissions do not necessarily dominate ambient PM burdens.

Oxidised organic PM contributed an additional 13–33%, which could not be directly attributed to a specific source but likely reflects a mixture of traffic, aged cooking emissions, and combustion. These fractional contributions should therefore be considered lower bounds. Our measurements also underscore the importance of secondary inorganic PM (e.g. ammonium nitrate and ammonium sulphate), which dominates PM concentrations in London and Beijing, accounting for 55–62% of observed mass. These components are formed through regional secondary processes from precursor emissions (NH₃, NOₓ and SO₂), indicating that a substantial fraction of PM in these developed megacities is formed through regional secondary processes rather than emitted directly, suggesting that local abatement measures alone may be insufficient to meet WHO guidelines. In contrast, PM mass in Delhi is predominantly organic, implying that targeted reductions in traffic and combustion emissions could still yield substantial improvements in air quality and public health. However, this organic fraction is not purely local, with a significant contribution also arising from advected pollution, including regional agricultural burning, which can episodically dominate PM levels^58^.

While our measurements provide chemically resolved insights into non-refractory PM_1_ emissions and concentrations, they do not capture refractory components such as brake wear, tyre abrasion, and road dust, meaning the total contribution from traffic-related emissions is underestimated. Nonetheless, the data highlight the evolving nature of urban PM sources, particularly in city centres where traditional combustion emissions are being gradually reduced—a trend set to continue on the road to Net Zero as vehicle fleets transition towards electric. As cities implement stricter controls on traffic and industrial emissions, addressing cooking-related emissions—through improved ventilation, filtration, and policy—may offer a practical and impactful route to further reduce urban PM exposure and improve public health.

## Methods

### Eddy covariance flux measurements

Chemically resolved aerosol fluxes (*F*_*c*_) can be calculated using the eddy covariance method which combines fast measurements (5 Hz) of the vertical wind velocity (*w*) with measured scalar concentrations (*c*) to yield a spatially averaged surface exchange flux (*F*_*c*_), as

1 $${F}_{c}(\varDelta t)=\overline{{w}{'} c{'} }.$$

Here, primes denote instantaneous fluctuations about the mean (e.g. ${w}^{{\prime} }=w-\,\bar{w}$), over bars denote block averages ( ~ 30 minutes) and *Δt*, represents the time lag that exists between the measurements of *w* and *c* due to the spatial separation of the sonic anemometer and particle analyser. At each site a high resolution aerosol mass spectrometer (HR-AMS) (Aerodyne Research Inc. Boston, MA)^59,60^ provided fast (5 Hz) measurements of PM_1_ aerosol composition (organics, nitrate, ammonium, sulphate and chloride)^35,36^ and a single particle soot photometer (SP2)^40,41,61^ was used to measure black carbon at the same sampling frequency (5 Hz).

### Flux quality assurance and uncertainty assessment

Flux quality assurance procedures are described in detail within the Supplementary Information. Briefly, time lags between vertical wind velocity and aerosol measurements were determined from campaign-average cross-covariance functions (Fig. S7), species-specific limits of detection were calculated following Langford et al. (2015) (Figs. S8–S12), and obvious outliers were removed prior to averaging. Conventional stationarity tests, turbulence quality assessments and friction velocity thresholds were not applied because chemically resolved aerosol fluxes measured by AMS often exhibit relatively low signal-to-noise ratios, such that these filtering approaches can preferentially remove small fluxes and introduce a systematic positive bias into ensemble averages. Instead, all valid fluxes were retained and uncertainty quantified through detection-limit analysis and sensitivity testing. Potential high-frequency attenuation and storage effects were also assessed. Storage corrections were generally small for organic aerosol fluxes (Table S1), while high-frequency losses were expected to be minor due to the elevated urban measurement heights and the turbulent scales sampled. Further details are provided in Section S5 of the Supplementary Information.

### Source apportionment of PM emissions

Positive Matrix Factorization (PMF) is a widely used receptor modelling technique for identifying and quantifying the sources of ambient aerosol. It works by decomposing a matrix of observed data—typically mass spectra—into a set of factor profiles and their associated time series, under the constraint that all values are non-negative. This reflects the physical reality that concentrations cannot be negative and helps ensure that the resulting factors are interpretable. PMF has become a standard tool for analysing organic aerosol data from aerosol mass spectrometers (AMS), with detailed methodologies described elsewhere^39,62^.

However, applying PMF to flux measurements presents a new challenge: fluxes can be both positive (emission) and negative (deposition), which violates the non-negativity assumption. Although the algorithm can be adapted to relax this constraint, direct application to bi-directional flux data often produces unrealistic and poorly constrained results. This typically appears as a small net flux being expressed as the sum of two factors, one with large emission fluxes offset by another with similarly large deposition fluxes, both of which lack physical meaning.

Previous applications of PMF to flux data—limited to gas-phase volatile organic compounds—have addressed this issue by applying PMF only to positive net fluxes^63,64^. However, this approach ignores deposition, which is essential for understanding net exchange. Or were derived from only a few masses, then not quantitatively describing the flux of total aerosol mass^28^.

In this study, we introduce a novel method for applying PMF indirectly to flux data by calculating organic aerosol fluxes using a numerical implementation of the “true” eddy accumulation (TEA) model rather than conventional eddy covariance. TEA is a micrometeorological technique originally proposed by Desjardins^65^ to quantify scalar fluxes (e.g., c_PM₁_) when sensors fast enough for eddy covariance are unavailable. Instead of sampling scalar concentrations at high frequency ( > 5 Hz), TEA separates air parcels transported by upward (w′↑) and downward (w′↓) moving eddies. Provided that sampling is proportional to the magnitude of the vertical wind velocity, the flux can be calculated directly as the difference in concentration between the updraft and downdraft reservoirs, multiplied by a scaling factor.

2 $${F}_{{PM}_{1}}=\overline{{w}^{\prime} {c}^{\prime}}_{{PM}_{1}} = \left(\frac{(\sum {|w}^{\prime} \uparrow {|{c}_{{PM}_{1}}}\uparrow )}{\sum {|w}^{\prime} \uparrow |} - \frac{(\sum {|w}^{\prime} \downarrow |{c}_{{PM}_{1}}\downarrow )}{\sum {|w}^{\prime} \downarrow |}\right)\left(\bar{\frac{{|w}^{\prime} |}{2}}\right)$$

This approach is challenging to implement in practice as it requires precise control of the sampling flow rate into up- and down-draught reservoirs at several Hertz, across a wide dynamic range reflecting the magnitude of |w′|. However, when vertical wind speed and scalar concentration data are available at sufficiently high temporal resolution, the TEA equation offers an alternative method for calculating fluxes that is numerically equivalent to eddy covariance. By virtually separating the data into theoretical up- and down-draught reservoirs, two positive aerosol concentrations (µg m⁻³) are obtained for each flux averaging period. These form a continuous time series alternating between (weighted) up- and down-draught concentrations, to which the PMF algorithm can be applied using standard protocols. Once an optimal PMF solution is identified, the concentration factors can be converted into flux factors by subtracting the down-draught factor concentrations from the up-draught values and multiplying the result by the TEA scaling factor, yielding fluxes in units such as ng m⁻² s⁻¹.

The weighted separation of organic aerosol concentrations into up and down draught components means a similarly weighted error is required. Here, we adopt the error model of Allan et al.^66^

3 $${\sigma }_{{diff}}=\alpha \sqrt{\frac{{I}_{o}+{I}_{c}}{\varDelta t}},$$

where α is a correction factor used to account for the random variation in the area of single-ion signals (α = 1.2), *I*_*o*_ is the ion signal when the chopper is open and *I*_*c*_ is the ion signal when the chopper is closed in Hz. The weighting of up and down draught errors is done so following Eq. 4 for up draught errors and Eq. 5 for down draught errors, which is consistent with the weighting applied to the concentration data in Eq. 2.

4 $$\uparrow {\sigma }_{{diff}}=\sqrt{\frac{{(\sum {|w}{\prime} \uparrow |{\sigma }_{{diff}}\uparrow )}^{2}}{\sqrt{(N\uparrow )\,\sum {|w}{\prime} {|}}}}\,$$

5 $$\downarrow {\sigma }_{{diff}}=\sqrt{\frac{{(\sum {|w}{\prime} \downarrow |{\sigma }_{{diff}}\downarrow )}^{2}}{\sqrt{(N\downarrow )}\sum {|w}{\prime} |}}\,$$

PMF solutions for each site are shown in Figs. S13–S33 of the Supplementary Information.

### Measurements of cooking PM

The overall efficiency with which the HR-AMS measures organic aerosols depends on both the collection efficiency (CE) and the ionization efficiency (RIE), typically referenced to nitrate^67^. Standard values used for organic PM are an RIE of 1.4 and a CE of 0.5, the latter reflecting particle losses due to bounce on the vaporizer. These defaults were applied in the measurements of cooking PM that underpinned the modelling study by Ots et al.^46^. More recent comparisons between filter-based and AMS measurements suggest that the AMS may overestimate cooking PM concentrations when using these default values, and that the CE for cooking aerosols is likely closer to unity. Laboratory studies have also shown that the RIE for cooking emissions can exceed 1.4, with reported values ranging from 1.5 to 3.5^47^. Similar findings were reported by Katz et al.^68^ for indoor measurements of cooking aerosol, who recommend applying an RIE of 1.5 for urban background sites and 2 for locations influenced by fresh cooking emissions. Accordingly, we calculated cooking aerosol concentrations and fluxes using a CE of 1 and an RIE of 2 across all three measurement sites. To reflect uncertainty in the RIE, we also report upper and lower bounds based on RIE values of 1.5 and 2.5, respectively.

### Cooking aerosol emission factors

Cooking aerosol emission rates (expressed in mg person⁻¹ day⁻¹) were estimated for each city on a per capita basis by dividing the average daily emission flux by the workday population density. For London and Beijing, where seasonal flux measurements were available, the daily emission rates were weighted to reflect seasonal representativeness—summer measurements were assumed to represent eight months of the year, and winter measurements the remaining four.

In London, the workday population density was calculated using the 2021 census data for Westminster, with a workday population of 367,508 and a borough area of 21.48 km², yielding a density of 17,109 people/km². For Beijing, population data were taken from Ma et al. (2017)^69^ for the Beijing Olympic Park area. In Delhi, estimates were based on the New Delhi District population of 1,173,902 (2011 census, Demography | District New Delhi, Government of NCT of Delhi | India), normalized by the district area of 42.73 km².

Uncertainties in the emission factors reflect the range of relative ionization efficiencies applied to cooking organic aerosol (COA), typically between 1.5 and 2.5, and a ± 10% uncertainty in population estimates.

## Supplementary information

NCASLangford_et_al_2026_SI_Revised_clean.

## Data Availability

Data supporting all figures are available at Figshare: 10.6084/m9.figshare.32626185.
