# Supplementary material for: Traffic and combustion emission controls leave cooking as a dominant local source of primary PM1 in megacities
Source: NPJ Clim Atmos Sci. 2026 Oct 23;9(1):212. doi: 10.1038/s41612-026-01583-6 (PMC13719519; doi:10.1038/s41612-026-01583-6)
Supplement: Supplementary file 1 [file 41612_2026_1583_MOESM1_ESM.docx]

**Supplementary Information for:**

**Traffic and combustion emission controls leave cooking as a dominant local source of primary PM_1_ in megacities**

Ben Langford^a*^, Chiara, F. Di Marco^a^., James M. Cash^a^., James D. Allan^b,c^., Zixuan Cheng^b^, Hugh Coe^b,c^., Delphine K. Farmer^d^., Pingqing Fu^e†^ ., Ranu Gadi^f^., **Bhola Ram Gurjar^g^**., Jose L. Jimenez^,h^., Joel Kimmel^i,j^., Dantong Liu^k^ ., Tuhin Kumar Mandal^l^., Neil J.Mullinger^a^., Gavin J. Phillips^m††^ ., Ernesto Reyes-Villegas^b†††^., Rutambhara Joshi^a††††^ Shivani Shivani^f^ and Eiko Nemitz^a^

^a^UK Centre for Ecology & Hydrology, Edinburgh Research Station, Bush Estate, Penicuik, EH26 0QB, United Kingdom

^b^School of Earth and Environmental Sciences, The University of Manchester, Manchester, UK

^c^National Centre for Atmospheric Science, The University of Manchester, Manchester, UK

^d^Department of Chemistry, Colorado State University, Fort Collins, CO 80523, United States

^e^Institute of Atmospheric Physics, Chinese Academy of Sciences, Beijing, China

**^f^Department of Applied Sciences and Humanities, Indira Gandhi Delhi Technical University for Women, Delhi, 110006, India**

^g^Department of Civil Engineering, Indian Institute of Technology Roorkee, India

^h^Cooperative Institute for Research in Environmental Sciences (CIRES) and Department of Chemistry, University of Colorado, Boulder, CO, USA

^i^TOFWERK AG., 3600 Thun, Switzerland

^j^Aerodyne Research Inc., Billerica, MA 01821, USA

^k^Zhejiang University, Hangzhou, China.

**^l^CSIR National Physical Laboratory, New Delhi, India**

^m^Department of Natural Sciences, University of Chester, Thornton Science Park, Chester, CH2 4NU, UK

^†^ Now at School of Earth System Science, Tianjin University, Tianjin, China

^††^ Now at Maastricht University, Maastricht, Limburg, NL

^†† †^ Now at Tecnologico de Monterrey, Escuela de Ingeniería y Ciencias, Av. General Ramon Corona 2514, Zapopan, CP 45138, Jalisco, Mexico.

^††††^Now at UK Centre for Ecology & Hydrology, Maclean Building, Wallingford, OX10 8BB, UK

* Corresponding Author: Ben Langford.

**Email:** [benngf@ceh.ac.uk](mailto:benngf@ceh.ac.uk)

# **S1 Site Description**

## *S1.1 London – BT Tower*

The city of London covers an area of 1710 km^2^ with 7.5 million inhabitants, while the wider London conurbation has a population of >13 million, making it the largest city in Western Europe. Measurements of submicron aerosol fluxes were made from the British Telecom (BT) tower (51°31′17.400″ N; 0°08′20.040″ W), a 190 m tall telecommunications tower, situated towards the NE of the city centre, at the east end of one of the major traffic axes (Marylebone Road) and to the SW of two major railway stations (King’s Cross and St. Pancras). The surrounding buildings have an average height of 15 m and comprise a mixture of commercial and residential buildings and according to the criteria of Stewart and Oke (1) the flux footprint of the tower has a local climate zone classification of 2 (compact high rise). A more detailed description of the measurement site, instrumentation used and typical flux footprint can be found within the REPARTEE campaign overview paper by Harrison et al. (2). Measurements took place during the summer (June-Aug) and winter (Dec-Jan) of 2021, with a total of 765 and 295 hours of flux data analysed for each measurement period, respectively.

During the winter campaign, the UK Government responded to the emergence of the Omicron variant of COVID-19 by issuing Plan B restrictive measures. These began on 8 December 2021 and included mandatory face coverings in most indoor public spaces, guidance to work from home where possible (from 13 December), and the requirement for NHS COVID Passes to attend large venues. Although hospitality venues remained open, the public were advised to limit social contact, which likelyreduced restaurant and pub attendance. Plan B measures were lifted on 27 January 2022 following a decline in case numbers and hospitalisations.

For the flux measurements a 3/8’’ OD copper inlet was mounted close to an ultrasonic anemometer (Solent R3-50, Gill Instruments, Lymington, UK) on top of a 15 m lattice frame, a permanent fixture at the top of the tower. At this location the influence of the building is small, with deflections of the wind flow caused by the tower found to between 0 and + 8° (3). The instruments were housed in a temporary laboratory space at 160 m. Air for analysis was pumped through the copper tube at a rate of > 60 l min^-1^, ensuring turbulent flow, and was subsampled by a high-resolution aerosol mass spectrometer (HR-AMS). To facilitate this the main inlet flow was split isokinetically using a TSI 4-way flow splitter, with two ports going to the main inlet pump, one going to a condensation particle counter and the other to the HR-AMS. The sample was dried prior to entering the HR-AMS using a drier (Perma Pure, MD-070-24S-2).

The HR-AMS allows submicron (< 1 µm) aerosol concentrations to be measured at a rate of up to 10 Hz which is sufficiently fast for direct eddy covariance flux measurements. The instrument has been routinely used to make aerosol composition measurements for well over a decade and has been described fully in the literature (4). Therefore, here we only focus on the specific sampling protocols used in this study. The HR-AMS was setup to alternate between standard concentration measurements and eddy covariance flux measurements in 30 minutes intervals. In flux mode, data were acquired at a rate of 10 Hz, with the chopper closed for the first and last 30 s of the half hour and open for the remaining 29 minutes, providing a 29-minute period of continuous data for eddy-covariance flux calculations within every hour.

*S1.2*	*Delhi – Indira Ghandi Delhi Technical University for Women*

Delhi is the largest megacity in India and the 5^th^ largest worldwide. It has an estimated population of >30 million and is considered one of the most polluted cities on Earth (5). Measurements were made as part of the Natural Environmental Research Council (NERC) funded Atmospheric Pollution and Human Health in an Indian Megacity programme. The measurement site was located in the north of the Old Delhi region at the Indria Ghandi Delhi Technical University for Women (IGDTUW) (28°39′51.800″ N; 77°13′55.200″ E). The university campus is situated between two busy roads, Lothian Road to the west and Mahama Gandhi Marg to the east. To the south of the campus is the Old Delhi railway station and to the north is Kashmere Gate, a very busy area with bus terminal. The prevailing wind direction was from the northwest/west, which covered a densely populated area consisting of a mix of domestic and commercial buildings. The average building height was ~10 m and the LCZ classification of the footprint was 2 (compact low-rise) (1).

A 30 m tall walkup tower was constructed on the eastern edge of the campus and a sonic anemometer was mounted to a mast at a height of 33 m above street level. Measurements were made by subsampling from a ½” O.D. (I.D. = 9.5 mm) copper inlet line which was approximately 50 m in length. The sample line was continually flushed at a flowrate of ~ 46 L min^-1^ to maintain turbulent flow. Sampled air was first (Perma Pure, MD-070-24S-2) before entering the HR-AMS.

Flux measurements were made between the 6^tth^ and 23^rd^ of November 2018, using the same protocol as used in London but with data acquired at a rate of 5 Hz. In total 288 hours of flux data were obtained and used in the analysis. More detailed information on the setup, operation and calibration of the HR-AMS can be found in Cash et al. (6). A single particle soot photometer (SP2, Droplet Measurement Technologies, Longmont, CO, US) sub-sampled (5 Hz), from the same inlet line, downstream of the HR-AMS, for direct measurements of black carbon concentrations and fluxes. The setup of the SP2 instrument has been described in detail by Joshi et al. (2021) (7).

*S1.3	Beijing – Institute of Atmospheric Physics*

Beijing has a population of >20 million and is the third largest megacity in the world. The city provides a distinct contrast to both London and Delhi, with a near absence of diesel emissions from within the city centre and larger electrification of the vehicle fleet at time of measurement. Whilst coal combustion within the city was officially prohibited in the city centre there was evidence of small-scale use e.g. of honeycomb coal briquettes for cooking. Coal is used in distance heating plants serving the city but located outside the flux footprint. Identifying and quantifying sources of air pollution within the city was the objective of theme I of the Atmospheric Pollution and Human Health (APHH) in a Chinese Megacity programme. As part of this work, direct flux measurements were made from the 325 m tall meteorological tower at the Institute of Atmospheric Physics (Chinese Academy of Sciences) (39°58′33.000″ N; 116°22′41.000″ E). The tower is located between the third and fourth city ring roads in a residential area, north of central Beijing. A detailed description of the site and the APHH programme is provided by Shi et al. (8).

A sonic anemometer (R3-50, Gill Instruments, Lymington, UK) was mounted to the tower at 102 m above street level. This was over twice the mean height (45 m) of the tallest residential buildings within the footprint, but still below the mixing layer height. The site has a local climate zone classification of 2 “compact mid-rise”, becoming LCZ1 “compact high-rise” to the southwest. Sample air was drawn from just below the sonic anemometer at a rate of ~90 L pm via a ½” O.D. copper tube (I.D. = 9.5 mm). This flow was split using a 4-way TSI flow splitter, to allow both the HR-AMS and SP2 to measure the same flow simultaneously, with air being dried before entering the instrument (Perma Pure, MD-070-24S-2). The HR-AMS was operated using the same sampling protocol as in London, but with data acquired at a rate of 5 Hz. Measurements took place during both winter (27/11/2016 – 09/12/2016) and summer (21/05/2017-26/06/2017) periods. In total 555 hours of flux data were collected in summer and 232 hours in winter. A detailed description of the black carbon flux measurements is provided by Joshi et al. (9).

**S2.	Concentrations**

Aerosol concentrations were derived from unit mass resolution data (m/z 12-340) collected by the Aerosol Mass Spectrometer (AMS) and processed using the Igor Pro-based software (Wavemetrics) SQUIRREL (Sequential Igor data Retrieval). Data were averaged to 30-minute intervals to match the time resolution of the flux measurements. Calibration followed the standard procedure of establishing the ionisation efficiency for nitrate by measuring the signal generated by ammonium nitrate particles of known size. Standard data analysis protocols were followed, including application of air beam corrections, relative ionisation efficiencies for the various chemical components, and composition-dependent collection efficiencies (CE). Where available, CEs were independently verified against external measurements e.g. PM filter analysis (Delhi), size resolved particle measurements (e.g. SMPS/CPC, Beijing, London)

The following plots show the average diurnal cycle for the five main species (Organics, Nitrate, Ammonium, Sulphate and Chloride), plus black carbon, measured during each of the measurement campaigns.

S2.1	London – Summer

**Figure S1. Diurnal variation in major PM₁ aerosol components above London during summer.** Average diurnal cycles of organics, nitrate, ammonium, sulphate and chloride measured using the aerosol mass spectrometer (AMS), together with black carbon (BC) measured using the single particle soot photometer (SP2). Data were averaged to 30-minute intervals to match the flux measurement resolution. Colours correspond to the components shown in the legend.

S2.2	London – Winter

**Figure S2. Diurnal variation in major PM₁ aerosol components above London during winter.** Average diurnal cycles of organics, nitrate, ammonium, sulphate and chloride measured using the aerosol mass spectrometer (AMS), together with black carbon (BC) measured using the single particle soot photometer (SP2). Data were averaged to 30-minute intervals to match the flux measurement resolution. Colours correspond to the components shown in the legend.

S2.3	Beijing – Summer

**Figure S3. Diurnal variation in major PM₁ aerosol components above Beijing during summer.** Average diurnal cycles of organics, nitrate, ammonium, sulphate and chloride measured using the aerosol mass spectrometer (AMS), together with black carbon (BC) measured using the single particle soot photometer (SP2). Data were averaged to 30-minute intervals to match the flux measurement resolution. Colours correspond to the components shown in the legend.

S2.4	Beijing Winter

**Figure S4. Diurnal variation in major PM₁ aerosol components above Beijing during winter.** Average diurnal cycles of organics, nitrate, ammonium, sulphate and chloride measured using the aerosol mass spectrometer (AMS), together with black carbon (BC) measured using the single particle soot photometer (SP2). Data were averaged to 30-minute intervals to match the flux measurement resolution. Colours correspond to the components shown in the legend.

S2.5	Delhi

**Figure S5. Diurnal variation in major PM₁ aerosol components above Delhi.** Average diurnal cycles of organics, nitrate, ammonium, sulphate and chloride measured using the aerosol mass spectrometer (AMS), together with black carbon (BC) measured using the single particle soot photometer (SP2). Data were averaged to 30-minute intervals to match the flux measurement resolution. Colours correspond to the components shown in the legend.

## **S3. Flux calculations**

Chemically resolved aerosol fluxes (*F_c_*) can be calculated using the eddy covariance method which combines fast measurements (2 Hz to 10 Hz depending on site) of the vertical wind velocity (*w*) with measured scalar concentrations (*c*) to yield a spatially averaged surface exchange flux (*F_c_*), following Eq. (S1).

$F_{c}(\Delta t)=\bar{w^{'}c'}$.	(S1)

Here, primes denote instantaneous fluctuations about the mean (e.g. $w^{'}=w- \bar{w}$ ), over bars denote block averages and *Δt*, represents the time lag that exists between the measurements of *w* and *c* due to the spatial separation of the sonic anemometer and HR-AMS.

### *S3.1 Background quantification and detrending*

In previous studies, the difference spectrum (open spectra minus closed spectra) for flux-mode data has typically been determined using a simple linear interpolation between the two closed periods (10, 11), as depicted in Figure S6A. However, our analysis indicates that this approach may be overly simplistic, especially for the analysis of fast-response data, as it fails to account for the slow vaporisation of certain compounds and their subsequent accumulation in the system.

In standard MS-mode, this phenomenon is not readily observed due to the regular and rapid switching between closed and open chopper cycles, typically every 5 to 15 seconds. In contrast, during flux-mode, the accumulative effect of slow vaporisation becomes clearly visible over the 29-minute open period. Figure S6 shows the open and closed signals for *m*/*z* 44 during flux-mode, which clearly demonstrates: (i) an exponential decay in the closed signal as material on the heater is slowly removed by vaporisation (Figure S6B), and (ii) an exponential rise to a maximum followed by a linear increase in the open signal.

As the AMS switches from standard MS-mode, where the particle beam is open 50% of the time, to a constantly open beam, the increased mass flux into the vaporisation region causes the instrument background to adjust to a higher value. There are two key reasons for correcting this behaviour: (a) Due to the non-linear time evolution, the effective mean background cannot be accurately derived by averaging the closed signals before and after the flux measurement period, which would lead to incorrect concentration estimates.
(b) Signal variations that do not reflect true variability caused by turbulent transport should be minimized as much as possible.

A high-pass filter based on linear detrending or a moving average is often applied prior to flux calculations. However, these methods are not suitable when the signal evolution is systematically non-linear. Since this behaviour was evident in many, though not all, m/z values, two independent methods were adopted to interpolate the background signal.

First, the extent of background accumulation was assessed for each m/z by calculating the increase in background (second closed period minus first closed period) as a percentage of the average open signal. If this increase was less than 5% of the average open signal, the closed signal was interpolated linearly between the two closed periods, following the approach of Farmer et al. (10).

In all other cases, the background closed signal was calculated using a two-stage process. First, a fit was applied to the 29-minute open data time series using the function:

$f\left( x \right)=y_{0}+Ae^{\left\{ \frac{-x}{\tau} \right\}}+mx$,	(S2)

This function describes an exponential rise to a maximum followed by a linear increase, as shown by the solid red line in Figure S6A. Here, *x* is time in seconds, *y₀* is the m/z signal at the transition point from exponential to linear behaviour, *τ* is the e-folding time of the exponential component, *A* is the amplitude of the exponential rise, and *m* is the slope of the linear section.

After deriving the best-fit coefficients to describe the non-linear evolution of the open signal, the value of *y₀* was adjusted to optimize the fit. The first and last points of the instrument background were then calculated using:

*y*_0_ = *I_c_ - A*	(S3)

where *I_c_* is the average of the first few data points (five in this case) from the second closed period. Substituting this adjusted *y₀* into Eq. (S2) yields the fitted background signal shown as the dashed red line in Figure S6A.

Figure S6A compares the fitted (red dashed line) and interpolated (black dashed line) backgrounds, while Figgure S6C shows the resulting difference signals (diff = open – closed). It is evident that failing to account for the slow vaporisation of compounds in flux mode can lead to a significant overestimation of the difference signal. This error could propagate into the flux measurements, although its impact may be mitigated depending on the averaging method used. For example, linear detrending is likely to reduce the error more effectively than block averaging.

**Figure S6. Correction of aerosol mass spectrometer background signals during flux-mode operation.** Raw aerosol mass spectrometer (AMS) signals during flux-mode operation. Grey shaded regions indicate periods when the chopper was in the closed position (the first and last 30 s of each 30 min averaging period), while unshaded regions indicate periods when the chopper was open. Panels (a) and (b) show temporal drift in both the open and closed signals caused by the slow vaporisation of material from the vaporiser. Panel (c) demonstrates that the difference signal (open minus closed) is biased high when the background is estimated by linear interpolation between the two closed periods. By contrast, estimating the background using Eq. S2 provides a more realistic correction of the open signal.

## *S3.2 Species fluxes*

Previous AMS measurements have presented flux measurements for individual mass-to-charge ratios (*m/z*) characteristic of particular chemical species (e.g. *m/z* 48 flux to represent sulphate or *m/z* 46 as nitrate), which were entirely attributed to the compound that dominates this *m/z*, thereby ignoring the potential contributions of other compounds (12-14). When working with the HR- AMS this problem can be avoided by integrating over pre-defined peak areas to target individual chemical ions, or using unit mass resolution data to calculate fluxes of the five main chemical species (10, 11). The later approach involves sub-dividing the mass spectra into chemical species through the application of the standard AMS fragmentation pattern. This method has been applied successfully by Farmer et al. (10, 11) but is not ideal because applying the fragmentation table to raw eddy covariance data (e.g., 2 to 10 Hz) propagates a higher level of noise into the derived species fluxes and is computationally expensive.

Here we present a new method, whereby fluxes of chemical species are calculated directly through the application of a flux fragmentation table which is akin to the fragmentation table used to derive standard species concentrations from the AMS (15). The flux of each unit mass resolution (UMR) *m/z* in the measured spectrum is calculated simultaneously yielding a flux mass spectra which may contain both positive (emission flux) and negative (deposition flux) values. This spectrum is subsequently multiplied by the flux fragmentation table to give the fluxes of the five main chemical species, organics, ammonium, nitrate, sulphate and chloride. The flux fragmentation table is a simplified version of the concentration fragmentation table. The major difference is that, for the flux calculation, contributions from gas phase compounds that do not yield a flux (e.g. O_2_, N_2_ etc.) thus do not need to be corrected for. Gas phase contributions from H_2_O and CO_2_, which do exhibit a mass flux, have been shown to be minor (typically less than 0.5 %) and are therefore not corrected (11). In the case of H_2_O, mass flux errors are further minimised by passing the sample air through a drier (Perma Pure, MD-070-24S-2) prior to entering the AMS.

Calculating the fluxes in this way is much less computationally expensive than applying the concentration fragmentation table to the raw eddy covariance data prior to the flux calculation, while maintaining the advantage of utilizing all the information held within the UMR mass spectra. Also, because the calculations are done on 29-minute average data rather than 2-10 Hz raw data, the new method is less likely to propagate noise between the compounds. For each data set, ions in the range of m/z 12-340 were used in the species flux calculations.

### *S3.3 Analysis of time-lags*

The spatial separation between the sonic anemometer and HR-AMS leads to a time-lag between the data recorded by the two instruments which must be accounted for in the post-processing of the data. There are various methods available to account for this time-lag. We have previously shown that the quantification of the time-lag by searching for the maximum cross-correlation within a prescribed time-lag window for flux measurements of trace gases and aerosols can lead to large biases where the signal-to-noise ratio is limited, as is the case for some of the m/z measured by the AMS (15). This effect can be avoided by using a prescribed time-lag (if justifiable) or by determining the time-lag through independent means. For this study, a prescribed time-lag for each dataset was determined by taking the campaign average time-lag, which was calculated by searching for a maximum in a cross-covariance function between m/z 46’ (nitrate) and w’ for each averaging period of the campaign. Figure S7 shows a histogram of time-lags measured at each of the four datasets. Time-lags of 2.5, 7 and 13.5 seconds were used for London, Delhi and Beijing, respectively.

**Figure S7. Distribution of aerosol flux time lags determined from cross-covariance analysis.** Histograms of the average time lags between the nitrate signal (m/z 46) measured by the aerosol mass spectrometer (AMS) and vertical wind velocity, determined from cross-covariance analysis for London (a), Delhi (b), and Beijing (c).

### *S3.4 Flux detection limits*

For trace gas and aerosol fluxes, detection limits are frequently determined based on the statistical properties of a cross-covariance function between vertical wind velocity (w) and scalar concentration (c). Characterising the general noise of the cross-covariance function at a distance well away (typically between −180 to −150 s and +150 to +180 s) from the true time lag by either taking the standard deviation (Langford et al., 2015(16)) or the root mean squared deviation from zero gives an estimate of the random error in the flux. Multiplying this value by a scaling factor, α, gives a basic, averaging-period-specific detection limit at a given confidence interval (e.g. 95th or 99th percentiles). This random error accounts for two effects: (a) statistical limitations of providing a representative picture of the turbulence through single-point measurements and (b) random noise in the concentration time series, which is due to instrument noise and, in the case of aerosol flux measurements, further exacerbated by the fact that the total signal is effectively quantised according to the number of particles entering the instrument.

Species-specific detection limits were determined by first calculating the root mean squared deviation from zero for each m/z (as described above, e.g. Langford et al., 2015(16)) and then applying the flux fragmentation table to the resulting errors. Fluxes falling below these limits were typically restricted to nighttime periods and were not removed, as their exclusion would only introduce a positive bias to the data when averaged. Instead, when averaging data to show diurnal patterns, we also averaged the individual LoD following the method of Langford et al. (2015) to ensure the final products were statistically significant. Figures S3 to S7 show the average diurnal pattern of fluxes measured above London, Delhi and Beijing, respectively. The shaded areas represent the averaged limit of detection at the 95th confidence interval. Where the shading intersects the zero line, the measured flux is not significantly different from zero.

Limits of detection are reduced when more data is available for averaging. In addition, flux detection limits were further improved in the more recent London measurements, where the AMS had been upgraded from the standard AP240 data acquisition card to the newer ADQ1600. The ADQ1600 offers higher bit depth and faster sampling, which enhance signal resolution and allow more effective noise suppression, improving sensitivity to low-abundance species leading to lower flux detection limits.

**Figure S8. Average aerosol fluxes measured above central London during summer relative to the estimated flux detection limits.** Average diurnal fluxes of organic aerosol (a), nitrate (b), ammonium (c), sulphate (d) and chloride (e) measured above central London during winter using the aerosol mass spectrometer (AMS). Shaded regions indicate the average flux detection limit at the 95th confidence interval, calculated following the method of Langford et al. (2015). Where the shaded region intersects the zero line, the measured flux is not significantly different from zero. Fluxes are shown as campaign-average diurnal cycles calculated from 30 min measurements.

**Figure S9. Average aerosol fluxes measured above central London during winter relative to the estimated flux detection limits.** Average diurnal fluxes of organic aerosol (a), nitrate (b), ammonium (c), sulphate (d) and chloride (e) measured above central London during winter using the aerosol mass spectrometer (AMS). Shaded regions indicate the average flux detection limit at the 95th confidence interval, calculated following the method of Langford et al. (2015). Where the shaded region intersects the zero line, the measured flux is not significantly different from zero. Fluxes are shown as campaign-average diurnal cycles calculated from 30 min measurements.

**Figure S10. Average aerosol fluxes measured above old Delhi relative to the estimated flux detection limits.** Average diurnal fluxes of organic aerosol (a), nitrate (b), ammonium (c), sulphate (d) and chloride (e) measured above central London during winter using the aerosol mass spectrometer (AMS). Shaded regions indicate the average flux detection limit at the 95th confidence interval, calculated following the method of Langford et al. (2015). Where the shaded region intersects the zero line, the measured flux is not significantly different from zero. Fluxes are shown as campaign-average diurnal cycles calculated from 30 min measurements.

**Figure S11. Average aerosol fluxes measured above Beijing during winter relative to the estimated flux detection limits.** Average diurnal fluxes of organic aerosol (a), nitrate (b), ammonium (c), sulphate (d) and chloride (e) measured above central London during winter using the aerosol mass spectrometer (AMS). Shaded regions indicate the average flux detection limit at the 95th confidence interval, calculated following the method of Langford et al. (2015). Where the shaded region intersects the zero line, the measured flux is not significantly different from zero. Fluxes are shown as campaign-average diurnal cycles calculated from 30 min measurements.

**.**

**Figure S12. Average aerosol fluxes measured above Beijing during summer relative to the estimated flux detection limits.** Average diurnal fluxes of organic aerosol (a), nitrate (b), ammonium (c), sulphate (d) and chloride (e) measured above central London during winter using the aerosol mass spectrometer (AMS). Shaded regions indicate the average flux detection limit at the 95th confidence interval, calculated following the method of Langford et al. (2015). Where the shaded region intersects the zero line, the measured flux is not significantly different from zero. Fluxes are shown as campaign-average diurnal cycles calculated from 30 min measurements.

*S3.5 Averaging approach and treatment of outliers*

To preserve the integrity of the diurnal signal and avoid systematic biases, we adopt the averaging approach described by Langford et al. (2015) (16), retaining all valid flux measurements regardless of whether they fall below the detection limit. This is particularly important for species subject to low signal-to-noise ratios (SNR), where individual fluxes may be indistinguishable from zero but ensemble averages can still reveal statistically significant patterns. Rather than applying friction velocity (*u*_*_) thresholds or stationarity filters, which are common in eddy covariance studies of high-SNR species, we retain the full dataset to ensure representativeness across the full diurnal cycle.

However, obvious outliers — defined as flux values that are inconsistent with surrounding data or exceed physically plausible bounds — were removed prior to averaging. These were typically identified through visual inspection and statistical screening. Averaging was performed across multiple days and by time of day, with the corresponding detection limits also averaged to ensure that the resulting fluxes were statistically robust. This approach is particularly suited to aerosol fluxes, where quantised signals and instrument noise can dominate individual measurements, but meaningful exchange patterns emerge when data are aggregated.

# **S4. Positive Matrix Factorization and Factor Fluxes**

The AMS data were pre-processed following Eq. 2 of the main manuscript such that each 30-minute flux averaging period was split into two (weighted) concentration values representing the updraft and downdraft components of the vertical wind velocity (e.g. the updraft was assigned to the hour mark and the downdraft to the half-hour). This transformation allows the PMF algorithm to operate on strictly positive values, as required, while preserving the flux-relevant structure of the data. Factor fluxes were subsequently derived by subtracting consecutive rows (i.e. updraft minus downdraft) for each of the concentration-based factors. Scaling by the standard deviation of the vertical wind velocity then transforms the resulting values into fluxes, with either positive (emission) or negative (deposition) sign.

As with standard application of PMF to AMS data, the PMF algorithm was applied iteratively to each dataset, increasing the number of factors until a solution was found that (i) minimised the Q/Qexp ratio and (ii) yielded factors that could be reasonably attributed to known sources. In each case, the selected solution was tested further by adding an additional factor to assess whether it improved the fit. However, this consistently led to “factor splitting”, indicating that the optimal number of factors had already been reached.

Once an appropriate number of factors had been identified, the solution was run ten times with different initialisation seeds to assess the robustness of the results. The standard deviation across these runs is shown as error bars in the subsequent figures. To explore rotational ambiguity, each solution was further examined using FPEAK values ranging from -1 to 1 in increments of 0.2. Only those solutions that remained stable across the range of FPEAK values were selected for further analysis.

Factors were assigned using a combination of characteristic AMS mass spectral signatures, temporal behaviour, diurnal cycles and comparison with previously published AMS source profiles. Traffic factors were identified by hydrocarbon-like organic aerosol spectra with prominent contributions from *m/z* 41, 43, 55 and 57 consistent with previously reported traffic factors. Solid-fuel and combustion factors were identified by prominent biomass-burning markers, particularly *m/z* 60 and 73, together with their temporal behavior and seasonal enhancement. Cooking aerosol factors were identified by strong contributions at *m/z* 55 together with elevated signals at *m/z* 67, 79, 81 and 91, combined with pronounced lunchtime and evening peaks corresponding to meal preparation periods. Oxidised organic aerosol factors were distinguished by increasing contributions from oxygenated fragments, particularly *m/z* 44, indicative of progressively aged organic material.

## *S4.1 London - Summer*

For the London datasets, PMF analysis did not consistently resolve the traffic-related factor. We therefore applied the ME-2 model to both datasets, constraining the traffic factor to the hydrocarbon-like organic aerosol (HOA) mass spectral profile previously characterised at the same site using the same instrument during the 2007 REPARTEE campaign (17). An alpha value of 0.1 was used, effectively anchoring the first factor to the reference spectrum within ±10%. All other factors were resolved without any a priori constraints.

The optimal London solution comprised five factors attributed to traffic, combustion, cooking, and two oxygenated components. These correspond to the less oxidised (Oxidised 1; semi-volatile) and more oxidised (Oxidised 3; low-volatility) ends of the oxygenated aerosol spectrum identified across all three city datasets, with the intermediate component (Oxidised 2) not resolved in London.

**Figure S13. ME-2 source profiles for the London summer dataset.** Mass spectral profiles derived from the ME-2 positive matrix factorisation (PMF) analysis of the London summer dataset using seed 0. Error bars show the standard deviation of solutions obtained using different initialisation seeds. Red shading indicates the 10% constraint applied to the traffic factor during the ME-2 analysis.

**Figure S14. Time series of ME-2 source factors for the London summer dataset.** Time series of the five factors identified by the ME-2 positive matrix factorisation (PMF) analysis of the London summer dataset.

**Figure S15. Flux diels of ME-2 source factors for the London summer dataset.** Average diurnal fluxes of the traffic (a), cooking 1 (b), cooking 2 (c), oxidised 1 (d) and oxidised 3 (e) factors identified by the ME-2 positive matrix factorisation (PMF) analysis of the London summer dataset. Error bars show ±1 standard deviation across the different initialisation seeds. Shaded regions indicate ±1 standard deviation of the hourly mean fluxes from the seed 0 solution.

**Figure S16. Concentration diels of PMF source factors for the London summer dataset.** Average diurnal concentrations of the traffic (a), cooking 1 (b), cooking 2 (c), oxidised 1 (d) and oxidised 3 (e) factors identified by the positive matrix factorisation (PMF) analysis of the London summer dataset. Error bars show ±1 standard deviation across the different initialisation seeds. Shaded regions indicate ±1 standard deviation of the hourly mean concentrations from the seed 0 solution.

*S4.2	London - Winter*

**Figure S17. ME-2 source profiles for the London winter dataset.** Mass spectral profiles derived from the ME-2 positive matrix factorisation (PMF) analysis of the London winter dataset using seed 0. Error bars show the standard deviation of the solutions obtained using different initialisation seeds. Red shading indicates the 10% constraint applied to the traffic factor during the ME-2 analysis.

**Figure S18. Time series of ME-2 source factors for the London winter dataset.** Time series of the four factors identified by the ME-2 positive matrix factorisation (PMF) analysis of the London winter dataset. Panels show the temporal variability of the resolved factor contributions throughout the measurement period.

**Figure S19. Flux diels of ME-2 source factors for the London winter dataset.** Average diurnal fluxes of the traffic (a), cooking (b), combustion (c) and oxidised 3 (d) factors identified by the ME-2 positive matrix factorisation (PMF) analysis of the London winter dataset. Error bars show ±1 standard deviation across the different initialisation seeds. Shaded regions indicate ±1 standard deviation of the hourly mean fluxes from the seed 0 solution.

**Figure S20. Concentration diels of ME-2 source factors for the London winter dataset.** Average diurnal concentrations of the traffic (a), cooking (b), combustion (c) and oxidised 3 (d) factors identified by the ME-2 positive matrix factorisation (PMF) analysis of the London winter dataset. Error bars show ±1 standard deviation across the different initialisation seeds. Shaded regions indicate ±1 standard deviation of the hourly mean concentrations from the seed 0 solution.

*S4.3	Beijing – Summer*

The PMF analysis of the Beijing data identified five distinct factors attributed to traffic, cooking and three aged organic aerosol factors (Oxidised 1, Oxidised 2 and Oxidised 3). The diurnal patterns of the cooking aerosol factor show the two peaks characteristic of cooking aerosol, which correspond to mealtimes, one at midday and the other in the early evening. Oxidised factor 2 shows a very similar diurnal pattern and it is likely that this represents oxygenated cooking aerosol.

**Figure S21. PMF source profiles for the Beijing summer dataset.** Mass spectral profiles derived from the positive matrix factorisation (PMF) analysis of the Beijing summer dataset using seed 0. Error bars show the standard deviation of the solutions obtained using different initialisation seeds.

**Figure S22. Time series of PMF source factors for the Beijing summer dataset.** Time series of the five factors identified by the positive matrix factorisation (PMF) analysis of the Beijing summer dataset. Panels show the temporal variability of the resolved factor contributions throughout the measurement period.

**Figure S23. Flux diels of PMF source factors for the Beijing summer dataset.** Average diurnal fluxes of the oxidised 3 (a), oxidised 2 (b), oxidised 1 (c), cooking (d) and traffic (e) factors identified by the positive matrix factorisation (PMF) analysis of the Beijing summer dataset. Error bars show ±1 standard deviation across the different initialisation seeds. Shaded regions indicate ±1 standard deviation of the hourly mean fluxes from the seed 0 solution.

**Figure S24. Concentration diels of PMF source factors for the Beijing summer dataset.** Average diurnal concentrations of the oxidised 3 (a), oxidised 2 (b), oxidised 1 (c), cooking (d) and traffic (e) factors identified by the positive matrix factorisation (PMF) analysis of the Beijing summer dataset. Error bars show ±1 standard deviation across the different initialisation seeds. Shaded regions indicate ±1 standard deviation of the hourly mean concentrations from the seed 0 solution.

*S4.4	Beijing – Winter*

The Beijing winter campaign was the most challenging data set to interpret using PMF and a solution could not be found that clearly separated out traffic, coal combustion and cooking factors. As with the London data set, the measurements were analysed using the ME-2 algorithm which allows a priori information to be used to anchor the solution for a specific factor to that of a reference spectrum. Here, we anchored factor 1 to that of the traffic factor identified in summer, allowing a variation of ±10 %. All other factors were unconstrained. With this approach, five factors were identified which were assigned to traffic, combustion, coal combustion, cooking and oxidized organic aerosol. The solution was run using 10 initialisation seeds, but there was no variation with seed and therefore these error bars are not shown in the subsequent plots.

**Figure S25. Factor contributions are insensitive to ME-2 initialisation seed for the Beijing winter dataset.** Stacked bar charts showing the fractional contribution of the five factors identified by the ME-2 positive matrix factorisation (PMF) analysis of the Beijing winter dataset across the 10 initialisation seeds. Factors were assigned to traffic, combustion, coal combustion, cooking and oxidised organic aerosol. No variation in factor contributions was observed between initialisation seeds, indicating a stable solution.

**Figure S26. ME-2 source profiles for the Beijing winter dataset.** Mass spectral profiles derived from the ME-2 positive matrix factorisation (PMF) analysis of the Beijing winter dataset using seed 0. Panels show the resolved source-factor mass spectra identified as traffic, combustion, coal combustion, cooking and oxidised organic aerosol. No variation was observed between the 10 initialisation seeds and therefore error bars are not shown.

**Figure S27. Time series of ME-2 source factors for the Beijing winter dataset.** Time series of the five factors identified by the ME-2 positive matrix factorisation (PMF) analysis of the Beijing winter dataset. Panels show the temporal variability of the resolved traffic, combustion, coal combustion, cooking and oxidised organic aerosol factors throughout the measurement period.

**Figure S28. Flux diels of ME-2 source factors for the Beijing winter dataset.** Average diurnal fluxes of the oxidised 3 (a), cooking (b), coal combustion (c), combustion (d) and traffic (e) factors identified by the ME-2 positive matrix factorisation (PMF) analysis of the Beijing winter dataset. Shaded regions indicate ±1 standard deviation of the hourly mean fluxes from the seed 0 solution. No variation was observed between the 10 initialisation seeds.

**Figure S29. Concentration diels of ME-2 source factors for the Beijing winter dataset.** Average diurnal concentrations of the oxidised 3 (a), cooking (b), coal combustion (c), combustion (d) and traffic (e) factors identified by the ME-2 positive matrix factorisation (PMF) analysis of the Beijing winter dataset. Shaded regions indicate ±1 standard deviation of the hourly mean concentrations from the seed 0 solution. No variation was observed between the 10 initialisation seeds.

*S4.5	Delhi*

The application of PMF to the Delhi dataset yielded a seven-factor solution, with the identified factors consistent with a high resolution analysis of concentrations made using the same dataset by Cash et al (6). The factors were assigned to two traffic factors, two combustion factors, a cooking factor and two oxidized organic aerosol factors. A detailed discussion of the identified factors and the methods used is present by Cash et al (6)

**Figure S30. ME-2 source profiles for the Delhi dataset.** Mass spectral profiles derived from the ME-2 positive matrix factorisation (PMF) analysis of the Delhi dataset using seed 0. Panels show the resolved source-factor mass spectra identified by the ME-2 analysis. Error bars show the standard deviation of the solutions obtained using different initialisation seeds.

**Figure S31. Time series of ME-2 source factors for the Delhi dataset.** Time series of the seven factors identified by the ME-2 positive matrix factorisation (PMF) analysis of the Delhi dataset. Panels show the temporal variability of the resolved source-factor contributions throughout the measurement period. Colours correspond to the source factors shown in the legend.

**Figure S32. Flux diels of ME-2 source factors for the Delhi dataset.** Average diurnal fluxes of the oxidised 3 (a), oxidised 2 (b), cooking (c), combustion 2 (d), combustion 1 (e), traffic 2 (f) and traffic 1 (g) factors identified by the ME-2 positive matrix factorisation (PMF) analysis of the Delhi dataset. Error bars show ±1 standard deviation across the different initialisation seeds. Shaded regions indicate ±1 standard deviation of the hourly mean fluxes from the seed 0 solution.

**Figure S33. Concentration diels of ME-2 source factors for the Delhi dataset.** Average diurnal concentrations of the oxidised 3 (a), oxidised 2 (b), cooking (c), combustion 2 (d), combustion 1 (e), traffic 2 (f) and traffic 1 (g) factors identified by the ME-2 positive matrix factorisation (PMF) analysis of the Delhi dataset. Error bars show ±1 standard deviation across the different initialisation seeds. Shaded regions indicate ±1 standard deviation of the hourly mean concentrations from the seed 0 solution.

**S5.	Flux losses**

High-frequency flux loss corrections were not applied to the measurements above London, Delhi, or Beijing, due to the measurement context and data quality considerations. At all three sites, fluxes were recorded well above the urban canopy, where the dominant turbulent eddies are larger and slower. In such conditions, high-frequency fluctuations represent only a small component of the total flux.

The sampling rates used—10 Hz in London and 5 Hz in both Delhi and Beijing—were considered sufficient to resolve the relevant turbulent scales. Any attenuation of higher-frequency components is therefore expected to be negligible.

Additionally, the signal-to-noise ratio of high frequency aerosol data from the AMS is typically limited (see Figure 4, (16)), and applying high-frequency corrections under such conditions risks amplifying noise and artefacts rather than improving accuracy. To ensure consistency across campaigns and avoid introducing site-specific biases, uncorrected fluxes were reported, with the understanding that any flux loss due to high-frequency attenuation is minor relative to other sources of uncertainty.

**S6. Storage corrections**

Storage error assessments were conducted for each site using a simple, single-point correction factor, calculated as:

$S_{err}= \frac{\left( C_{2}-C_{1} \right)}{\left( t_{2}-t_{1} \right)} z$,	(S4)

where *C_2_* and *C_1_* are the concentrations at the current and previous time points, respectively, *t_2_* and *t_1_* are the corresponding timestamps, and *z* is the measurement height. The corrected flux (*F_corr_*) was then calculated as:

$F_{corr}=F+S_{err}$	(S5)

To evaluate the magnitude of storage effects, uncorrected and corrected PM₁ fluxes were compared using regression analysis. Table S1 lists the slope of the correction factor for each site. Organic aerosol fluxes were generally robust across sites and seasons, with slopes close to unity, indicating minimal bias from processes occurring below the measurement height within the urban boundary layer. However, London in winter exhibited a notable deviation (slope = 1.10), suggesting enhanced storage effects under stable atmospheric conditions. Given that London had the highest inlet height (190 m), it is possible that a shallow nocturnal boundary layer formed, leading to greater accumulation and larger storage errors. In contrast, inorganics such as ammonium and sulphate showed greater variability, particularly in summer at higher inlet heights, where thermodynamic partitioning below the measurement level led to apparent deposition artefacts or altered emission signals. Fluxes presented in the main manuscript are uncorrected for storage, to reflect the directly measured exchange and avoid introducing uncertainties associated with estimating sub-inlet accumulation from a single concentration measurement.

**Table S1.** **Slopes from regression analysis comparing uncorrected and storage-corrected PM₁ aerosol fluxes for each site and season.** Values indicate the magnitude of the storage correction, with slopes >1 suggesting net accumulation below the measurement height and slopes <1 indicating apparent deposition artefacts.

|  | **Organics** | **Nitrate** | **Ammonium** | **Sulphate** | **Chloride** |
| --- | --- | --- | --- | --- | --- |
| **London – Summer** | 1.00 | 1.10 | 0.94 | 0.93 | 1.00 |
| **London- Winter** | 1.10 | 1.05 | 1.01 | 0.96 | 1.08 |
| **Beijing – Summer** | 1.03 | 1.02 | 0.93 | 0.81 | 1.09 |
| **Beijing – Winter** | 0.98 | 0.91 | 1.00 | 0.98 | 0.99 |
| **Delhi** | 0.92 | 1.01 | 1.01 | 1.02 | 1.01 |

**References**

1.	I. D. Stewart, T. R. Oke, Local Climate Zones for Urban Temperature Studies. *Bulletin of the American Meteorological Society* **93**, 1879-1900 (2012).

2.	R. M. Harrison *et al.*, Atmospheric chemistry and physics in the atmosphere of a developed megacity (London): an overview of the REPARTEE experiment and its conclusions. *Atmospheric Chemistry and Physics* **12**, 3065-3114 (2012).

3.	C. R. Wood *et al.*, Turbulent Flow at 190 m Height Above London During 2006–2008: A Climatology and the Applicability of Similarity Theory. *Boundary-Layer Meteorology* **137**, 77-96 (2010).

4.	P. F. DeCarlo *et al.*, Field-deployable, high-resolution, time-of-flight aerosol mass spectrometer. *Analytical Chemistry* **78**, 8281-8289 (2006).

5.	W. A. Quality (2020) World Air Quality Report - Region & City PM2.5 Ranking. (IQAir), p 41.

6.	J. M. Cash *et al.*, Seasonal analysis of submicron aerosol in Old Delhi using high-resolution aerosol mass spectrometry: chemical characterisation, source apportionment and new marker identification. *Atmos. Chem. Phys.* **21**, 10133-10158 (2021).

7.	R. Joshi (2021) Black carbon flux measurements in megacities using the eddy covariance method. (The University of Manchester).

8.	Z. Shi *et al.*, Introduction to the special issue “In-depth study of air pollution sources and processes within Beijing and its surrounding region (APHH-Beijing)”. *Atmos. Chem. Phys.* **19**, 7519-7546 (2019).

9.	R. Joshi *et al.*, Direct measurements of black carbon fluxes in central Beijing using the eddy covariance method. *Atmos. Chem. Phys.* **21**, 147-162 (2021).

10.	D. K. Farmer *et al.*, Chemically Resolved Particle Fluxes Over Tropical and Temperate Forests. *Aerosol Sci. Technol.* **47**, 818-830 (2013).

11.	D. K. Farmer *et al.*, Eddy covariance measurements with high-resolution time-of-flight aerosol mass spectrometry: a new approach to chemically resolved aerosol fluxes. *Atmospheric Measurement Techniques* **4**, 1275-1289 (2011).

12.	E. Nemitz *et al.*, An eddy-covariance system for the measurement of surface/atmosphere exchange fluxes of submicron aerosol chemical species - First application above an urban area. *Aerosol Sci. Technol.* **42**, 636-657 (2008).

13.	R. Zalakeviciute *et al.*, Chemically-resolved aerosol eddy covariance flux measurements in urban Mexico City during MILAGRO 2006. *Atmospheric Chemistry and Physics* **12**, 7809-7823 (2012).

14.	M. Gordon *et al.*, Aerosol flux measurements above a mixed forest at Borden, Ontario. *Atmos. Chem. Phys.* **11**, 6773-6786 (2011).

15.	J. D. Allan *et al.*, Quantitative sampling using an Aerodyne aerosol mass spectrometer - 1. Techniques of data interpretation and error analysis. *Journal of Geophysical Research-Atmospheres* **108** (2003).

16.	B. Langford, W. Acton, C. Ammann, A. Valach, E. Nemitz, Eddy-covariance data with low signal-to-noise ratio: time-lag determination, uncertainties and limit of detection. *Atmospheric Measurement Techniques* **8**, 4197-4213 (2015).

17.	J. D. Allan *et al.*, Contributions from transport, solid fuel burning and cooking to primary organic aerosols in two UK cities. *Atmos. Chem. Phys.* **10**, 647-668 (2010).
